# Schmerzmanagement bei geriatrischen Patient:innen – Methodenpapier zur S3-Leitlinie „GeriPAIN“

**DOI:** 10.1007/s00482-025-00875-9

**Published:** 2025-03-27

**Authors:** Melina Hendlmeier, Thomas Fischer, Corinna Drebenstedt, Stephan Fuchs, Heike Norda, Erika Sirsch

**Affiliations:** 1 Deutsche Schmerzgesellschaft e. V., Alt-Moabit 101b, 10559 Berlin, Deutschland; 2 Evangelische Hochschule Dresden, Dresden, Deutschland; 3 St. Marien-Hospital, Friesoythe, Deutschland; 4 Institut für Allgemeinmedizin der Martin-Luther-Universität Halle-Wittenberg, Halle, Deutschland; 5 UVSD SchmerzLOS e. V., Neumünster, Deutschland; 6 Universität Duisburg-Essen, Duisburg-Essen, Deutschland

**Keywords:** Demenz, Sektorenübergreifende Zusammenarbeit, Geriatrie, Ältere Menschen, Interprofessionelle Zusammenarbeit, Dementia, Cross-sectional collaboration, Geriatrics, Older adults, Interprofessional Collaboration

## Abstract

Geriatrische Patient:innen zeichnen sich durch ein höheres Lebensalter und eine geriatrietypische Multimorbidität aus. Mit steigendem Lebensalter nimmt die Prävalenz von Schmerzen zu. Insbesondere bei den geriatrischen Patient:innen gehört Schmerz zu den häufig auftretenden Merkmalskomplexen. In Deutschland wird ein Trend beobachtet, dass über die letzten Jahre hinweg die Schmerzprävalenz und die Schmerzintensität bei älteren Menschen weiter zugenommen hat. Das Schmerzmanagement stellt bei dieser besonders vulnerablen Gruppe die Patient:innen selbst, ihre Angehörigen und professionelle Akteure im Gesundheitswesen vor große Herausforderungen. Daher wird die auslaufende S3-Leitlinie zum „Schmerzassessment bei älteren Menschen in der vollstationären Altenhilfe“ aufgegriffen, aktualisiert und zum Thema Schmerztherapie erweitert sowie auf den ambulanten und akutstationären Bereich ausgeweitet.

## Abstract (en)

Geriatric patients are characterized by an advanced age and typical geriatric multimorbidity. The prevalence of pain increases with age. In geriatric patients in particular, pain is one of the most frequently occurring characteristic complexes. In Germany, a trend has been observed that the prevalence and intensity of pain in older people has continued to increase in recent years. Pain management in this particularly vulnerable group poses major challenges for the patients themselves, their relatives and healthcare professionals. For this reason, the expiring S3 guideline on “Pain assessment in older people in full inpatient care for the elderly” will be taken up, updated and expanded to include pain therapy and extended to outpatient and acute inpatient care.

## Warum diese Leitlinie?

Für ältere Betroffene in stationären Pflegeeinrichtungen lassen sich akute und/oder chronische Schmerzprävalenzen von über 80 % (*N* = 239) nachweisen, wie sich in der Interventionsstudie von Dräger et al. (2017) zeigt [14]. Internationale Übersichtsarbeiten zeichnen ein ähnliches Bild [10, 13]. Bis zu 85 % der Menschen in stationären Pflegeeinrichtungen leiden demnach unter akuten und bis zu 58,1 % unter chronischen Schmerzen [10]. Chronische muskuloskelettale Schmerzen, Gelenkschmerzen und neuropathische Schmerzen sind die häufigsten Schmerzarten [13, 26]. Geriatrische Multimorbidität ist mit einer höheren Anzahl an Schmerzorten und höherer Schmerzintensität assoziiert [5]. Bei einem Teil dieser Betroffenen wird trotz Behandlung keine suffiziente Schmerzlinderung erreicht [15, 22]. Kognitive Beeinträchtigungen führen dazu, dass Schmerzen nicht mehr adäquat von den Betroffenen kommuniziert werden können. Das Vorhandensein mehrerer chronischer Erkrankungen, wie es bei geriatrischen Patient:innen der Fall ist, erschwert das Erkennen von schmerzbezogenen Symptomen. Mangelndes Wissen zu validierten Assessmentinstrumenten und Kommunikationsbarrieren durch hierarchisch organisierte Strukturen behindern ebenfalls eine angemessene Schmerzbeurteilung [20]. Das Schmerzassessment wie auch das Monitoring der Schmerztherapie gestaltet sich daher besonders herausfordernd.

Das Innovationsfondsprojekt „GeriPAIN“ (01VSF22017) widmet sich der Erstellung einer Leitlinie, die sich den Besonderheiten des Schmerzmanagements geriatrischer Patient:innen annimmt, vom Schmerzassessment bis zur Evaluation der Schmerztherapie. Ein besonderer Fokus wird auf der sektorenübergreifenden, interprofessionellen Zusammenarbeit liegen. Versorgungsbrüche sollen reduziert, möglichst vermieden werden. Die gemeinsame Entscheidungsfindung („shared decision-making“) mit den Betroffenen und ihren Angehörigen soll gestärkt werden. Neben der Leitlinie werden innerhalb des Projekts Qualitätsindikatoren sowie eine Patient:innenleitlinie entwickelt.

Adressat:innen der Leitlinie sind geriatrische Patient:innen mit und ohne kognitive Einschränkungen in allen Versorgungsarrangements. Die Leitlinie richtet sich daher an alle an der Versorgung beteiligten Berufsgruppen, wie z. B. Ärzt:innen, Pflegefachpersonen, Psycholog:innen, Physiotherapeut:innen, Ergotherapeut:innen und Sozialarbeiter:innen.

## Ziel der Leitlinie

In der Leitlinie werden die Besonderheiten des Schmerzmanagements bei geriatrischen Patient:innen in den Blick genommen, damit ist sie eine Ergänzung zum generellen Schmerzmanagement. Es werden die wissenschaftliche Fundierung verfügbarer Assessmentverfahren, schmerztherapeutische Ansätze sowie die Gestaltung der sektorenübergreifenden und interprofessionellen Zusammenarbeit gesichtet, bewertet und für die Versorgungspraxis interpretiert. Der gemeinsamen Entscheidungsfindung aller Beteiligten kommt eine besondere Bedeutung zu.

## Definition „geriatrischer Patient:innen“

Geriatrische Patient:innen sind charakterisiert durch eine geriatrietypische Multimorbidität **und** ein höheres Lebensalter [16, 23, 24]. Darunter wird das Vorliegen von mindestens zwei chronischen Erkrankungen mit sozialmedizinischer Relevanz im Sinne einer alltagsrelevanten Aktivitätsbeeinträchtigung verstanden. Zur Operationalisierung „geriatrietypischer Multimorbidität“ erarbeiteten die geriatrischen Fachgesellschaften Abgrenzungskriterien der Geriatrie [3]. Diese wurden in einer Analyse von Routinedaten der gesetzlichen Krankenversicherung im Wesentlichen als praktikabel bestätigt [21]. Geriatrietypisch sind unter anderem die in Tab. 1 aufgelisteten Schädigungen der Körperfunktionen und -strukturen entsprechend der International Classification of Functioning [6, 21, 23, 24].

**Tab. 1:** Wichtige geriatrietypische Schädigungen der Körperfunktionen und -strukturen^1^

Kognitive Defizite
Depression, Angststörung
Sturzneigung und Schwindel
Sensibilitätsstörungen
Herabgesetzte Medikamententoleranz
Inkontinenz (Harninkontinenz, selten Stuhlinkontinenz)
Störungen im Flüssigkeits- und Elektrolythaushalt
Dekubitalulzera
Fehl- und Mangelernährung
Herabgesetzte körperliche Belastbarkeit/Gebrechlichkeit

^1^In Anlehnung an: Medizinischer Dienst des Spitzenverbandes Bund der Krankenkassen e. V. (MDS) 2021, S. 75 [23]

Eine sozialmedizinische Relevanz besteht, wenn die beeinträchtigte Funktionsfähigkeit Einfluss auf die Teilhabe nimmt. Einschränkungen wirken sich damit direkt auf die persönliche Lebensgestaltung und das Lebensumfeld aus [29].

In einem höheren Lebensalter befinden sich in der Regel Menschen ab dem 70. Lebensjahr [16, 23]. Das Bundessozialgericht urteilte 2015, dass bei einer ausgeprägten geriatrietypischen Multimorbidität diese Altersgrenze bis auf das 60. Lebensjahr herabgesetzt werden kann [9].

Ab einem Lebensalter von 80 Jahren oder älter werden Patient:innen generell als geriatrisch betrachtet. Aufgrund altersphysiologischer Veränderungen sowie bestehender Schädigungen der Körperfunktionen und -strukturen sind die Reservekapazitäten eingeschränkt. Menschen ab dem 80. Lebensjahr sind daher besonders vulnerabel. Vulnerabilität bezeichnet in diesem Zusammenhang das regelhafte Auftreten a) von Komplikationen und Folgeerkrankungen, b) eines erhöhten Chronifizierungsrisikos und c) eines erhöhten Risikos eines Verlusts an Selbstbestimmung und selbstständiger Lebensführung [6, 16, 23].

## Finanzierung der Leitlinie

Die Erstellung der Leitlinie wird durch den Innovationsfonds des Gemeinsamen Bundesausschusses gefördert (Förderkennzeichen 01VSF22017). Die Konsortialführung hat die Deutsche Schmerzgesellschaft e. V. inne, Konsortialpartner ist die evangelische Hochschule Dresden. Die Deutsche Gesellschaft für Geriatrie e. V. und die Unabhängige Vereinigung aktiver Schmerzpatienten in Deutschland SchmerzLOS e. V. sind wesentliche Kooperationspartner sowie Mitantragstellende. Neben einer Teilzeitstelle für eine wissenschaftliche Mitarbeiterin und eine wissenschaftliche Hilfskraft wird die Projektleitung in geringem Umfang gefördert, jede weitere Mitarbeit an der Leitlinienentwicklung ist ehrenamtlich.

## Steuergruppe der Leitlinie

**Abb. 1:**
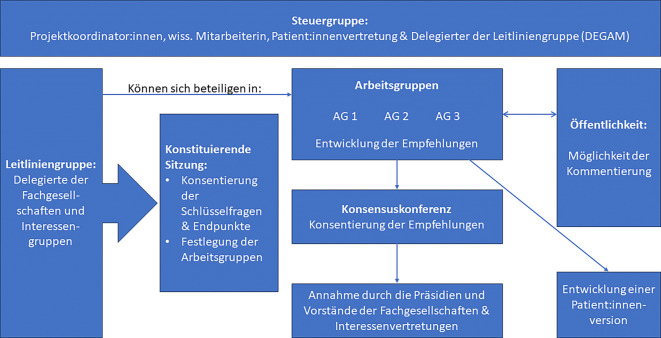
Beteiligte Personen im Ablauf der Leitlinienentwicklung

Die Steuergruppe besteht aus den Leitlinienkoordinator:innen, Delegierten der beteiligten Konsortial- und Kooperationspartner, der wissenschaftlichen Mitarbeiterin und einem Delegierten der Deutschen Gesellschaft für Allgemein- und Familienmedizin, der bei der konstituierenden Sitzung durch die Leitliniengruppe gewählt wurde (vgl. Abb. 1). Die inhaltliche und redaktionelle Verantwortung für die Empfehlungen der Leitlinie liegt bei der Steuergruppe.

## Beteiligte Fachgesellschaften und Interessengruppen

Anwendungsgruppen sollen möglichst umfassend einbezogen werden. Insgesamt sind 32 Fachgesellschaften und Interessenvertretungen sowie zwei Patient:innenvereinigungen an der Leitlinienentwicklung beteiligt und bilden somit das Spektrum des Teams in der geriatrischen Versorgung sowie der verschiedenen Settings breit ab. Weitere Detailinformationen können bei der AWMF e. V. eingesehen werden (AWMF-Registernummer 145-005).

## Interessenkonflikte

Zu Beginn der Leitlinienentwicklung legen alle beteiligten Personen ihre Interessenkonflikte offen [2]. Dazu wird das Onlineportal „Interessenerklärung Online“ der AWMF verwendet. Mögliche Interessenkonflikte werden durch die Mitglieder der Steuergruppe auf Relevanz und Unbedenklichkeit hin geprüft. Die Prüfung möglicher Interessenkonflikte der Steuergruppe erfolgt durch das Präsidium der Deutschen Schmerzgesellschaft e. V. Im abschließenden Methodenreport der Leitlinie werden – entsprechend dem durch die AWMF definierten Verfahren [2] – die Erklärungen zu den Interessenkonflikten der beteiligten Personen detailliert veröffentlicht.

## Beteiligung der Delegierten

Die Minimalvoraussetzung für eine Beteiligung der Delegierten als Mandatstragende ihrer Fachgesellschaften ist die Teilnahme an der Konsentierung der Gesamtleitlinie zum Abschluss. Alle Delegierten können sich an der inhaltlichen Entwicklung der Leitlinie durch die Arbeitsgruppen beteiligen (Abb. 1). Die jeweilige Beteiligung wird im abschließenden Methodenreport ausgewiesen.

Es bilden sich unterschiedliche Ebenen der Beteiligung heraus, zum einen Arbeitsgruppen, deren Mitglieder an der Bearbeitung der Literatur und der Erarbeitung der Empfehlungen beteiligt sind. Zum anderen die Ebene der Leitliniengruppe, bei der alle Delegierten die Schlüsselfragen, die Endpunkte und die Empfehlungen konsentieren.

## Arbeitsgruppen

Die Mitglieder der drei Arbeitsgruppen arbeiten zu folgenden Schwerpunkten:

- AG 1: Schmerzassessment (inkl. Diagnostik)
- AG 2: interprofessionelle und sektorenübergreifende Zusammenarbeit
- AG 3: Besonderheiten in der Schmerztherapie

Es werden regelmäßige gemeinsame Übungseinheiten zum Literaturscreening, zur Datenextraktion und zur methodischen Bewertung der Literatur angeboten, um den methodischen Kenntnisstand der Delegierten abzugleichen.

## Leitliniengruppe

In der Leitliniengruppe sind Delegierte der Fachgesellschaften und Interessenvertretungen vertreten. Sie trifft sich zum Abschluss der Leitlinienarbeit und konsentiert die durch die Arbeitsgruppen erarbeiteten Ergebnisse zu den Empfehlungen der Leitlinie. Für die Empfehlungen wird ein starker Konsens angestrebt (Tab. 2), Minderheitenvoten werden ausgewiesen. Vor der abschließenden Veröffentlichung muss die Leitlinie durch die jeweiligen Präsidien bzw. Vorstände angenommen werden.

**Tab. 2:** Feststellung der Konsensusstärke^2^

Starker Konsens	Zustimmung von > 95 % der Teilnehmenden
Konsens	Zustimmung von > 75–95 % der Teilnehmenden
Mehrheitliche Zustimmung	Zustimmung von > 50–75 % der Teilnehmenden
Keine mehrheitliche Zustimmung	Zustimmung von ≤ 50 % der Teilnehmenden

^2^Entnommen aus: AWMF 2020, S. 62 [2]

## Einbeziehung der Betroffenenperspektive

Die Perspektive der Betroffenen wird insbesondere durch die Delegierte von SchmerzLOS e. V. eingebracht, die Mitantragsstellende des Leitlinienvorhabens und daher in der Steuergruppe vertreten ist. Ebenso ist die Bundesarbeitsgemeinschaft der Seniorenorganisationen (BAGSO) e. V. beteiligt. Delegierte der Patient:innenvertretungen werden an unterschiedlichen Stellen der Leitlinienentwicklung beteiligt: Auswahl des Leitlinienthemas, Entwicklung der Schlüsselfragen, Suche und Auswertung der Evidenz, Entwicklung der Empfehlungen, Überarbeitung der Textfassung, Mitarbeit an der Patient:innenleitlinie und kontinuierliche Beteiligung an der Leitliniensteuerung [8].

## Methodisches Vorgehen

Das methodische Vorgehen orientiert sich am AWMF-Regelwerk Leitlinien [2] für die Erstellung von S3-Leitlinien und am Manual Systematische Recherche für Evidenzsynthesen und Leitlinien [4].

## Schlüsselfragen

Die Steuergruppe entwickelte zur konstituierenden Sitzung klinisch relevante Schlüsselfragen. Es wurden neun Schlüsselfragen aus der S3-Leitlinie „Schmerzassessment bei alten Menschen in der vollstationären Altenhilfe“ übernommen und um das Setting der ambulanten und akutstationären Versorgung ergänzt. Vier Schlüsselfragen zu Besonderheiten der Diagnostik und der Therapie bei geriatrischen Patient:innen mit Schmerzen wurden formuliert, zwei Schlüsselfragen zur gemeinsamen Entscheidungsfindung und der Aufrechterhaltung der Versorgungskontinuität erarbeitet. Die Schlüsselfragen wurden bei der konstituierenden Sitzung angepasst und durch die Delegierten der Leitliniengruppe konsentiert. Ziel ist es, zu möglichst allen Fragen eine Empfehlung zu formulieren.

## Literatursuchstrategie

Zunächst findet eine Suche zu (inter-)nationalen Leitlinien zu Schmerz über die Datenbank des Guideline International Network (G-I-N) statt. Identifizierte Quellleitlinien werden anschließend anhand des AGREE-II-Instruments [7] bewertet und eine Synopse erstellt. Neben dieser Suche erfolgt eine strukturierte Suche nach systematischen Übersichtsarbeiten in den Datenbanken Medline via PubMed, Cumulative Index to Nursing and Allied Health Literature (CINAHL) via EBSCO, Cochrane Database of Systematic Reviews und Embase via Elsevier.

Der von der Steuergruppe entwickelte Suchstring wird durch die Delegierten in den Arbeitsgruppen diskutiert und konsentiert.

## Bewertung der Ergebnisse der Literaturrecherche und Ausweisung der Evidenzstufen

Die Analyse der Leitlinien und der Übersichtsarbeiten wird durch die Software Covidence® [11] unterstützt. Das Vorgehen zur Identifikation geeigneter Übersichtsarbeiten orientiert sich am Prisma-Schema [27]. Es werden a priori Ein- und Ausschlusskriterien festgelegt. Die identifizierten Publikationen werden durch zwei unabhängige Mitglieder der Arbeitsgruppen zuerst anhand des Titels und Abstracts und im Anschluss anhand des Volltexts auf ihre Relevanz hin geprüft. Konflikte werden durch ein drittes Mitglied gelöst. Können nicht alle Fragestellungen durch die Quellleitlinien und systematischen Übersichtsarbeiten beantwortet werden, kann die Suche nach randomisierten, kontrollierten Studien bzw. bei Bedarf auch nach nichtrandomisierten, kontrollierten Studien ausgedehnt werden.

Die methodische Bewertung der eingeschlossenen Publikationen erfolgt anhand von strukturierten Bewertungsinstrumenten. Für systematische Übersichtsarbeiten von Interventionsstudien wird AMSTAR‑2 [30], für alle weiteren Übersichtsarbeiten das Instrument des Joanna Briggs Institute [19] angewendet. Die Bewertung erfolgt durch die wissenschaftliche Mitarbeiterin. Es werden 10 % der Studien zufällig ausgewählt und durch ein zweites Mitglied der Steuergruppe bewertet. Die inhaltliche Auswertung der Übersichtsarbeiten findet gemeinsam mit den Arbeitsgruppenmitgliedern statt.

Die Ausweisung der Evidenz erfolgt anhand der Oxford-Klassifikation von 2011 [18]. Das Studiendesign sowie dessen interne Validität bestimmen dabei die primäre Zuordnung zu einem „level of evidence“ (LoE) von 1 bis 5. Systematische Übersichtsarbeiten führen in der Regel zu einem LoE 1, je nach Art der zugrunde liegenden Fragestellung und eingeschlossenen Primärstudien. Eine Herabstufung des LoE ist möglich, z. B. bei hohem Verzerrungsrisiko oder Indirektheit [18].

## Handlungsempfehlungen der Leitlinie

Die Graduierung der Handlungsempfehlungen spiegelt wider, inwieweit erwünschte Konsequenzen gegenüber den negativen oder keinen Konsequenzen überwiegen [2]. Die Graduierung erfolgt in „starke“, „schwache“ und „offene“ Empfehlungen (soll – sollte – kann), ggf. können bei fehlender Evidenz Empfehlungen im Expertenkonsens ausgesprochen werden [28]. Sie basiert auf einer „Nutzen-Schaden Abwägung, dem Vertrauen in die identifizierte Evidenz – insbesondere in die Effektstärken-, den Ansichten und Präferenzen der betroffenen Patient:innen/Bürger:innen sowie der klinischen Expertise der Leitliniengruppe“ [2]. In der Regel ist eine starke Empfehlung für oder gegen eine Maßnahme an eine hohe Evidenz gekoppelt. Empfehlungen der Leitlinie können allerdings in begründeten Fällen davon abweichen, z. B. wenn Studienergebnisse, die zu Empfehlungen führen, herauf- bzw. herabgestuft werden [31].

## Externes Review und Geltungsdauer der Leitlinie

Der Leitlinienentwurf wird zu einer externen Konsultation für den Zeitraum von sechs Wochen auf der Homepage der Deutschen Schmerzgesellschaft e. V. öffentlich zugänglich sein. Expert:innen, Anwender:innen sowie interessierte Personen werden eingeladen, Rückmeldungen zu geben. Eine digitale oder eine offen formulierte schriftliche Rückmeldung per E‑Mail oder Post ist möglich. Im Anschluss an die Konsultationsphase werden Kommentare und Änderungsvorschläge durch die Steuergruppe geprüft und ggf. in Rücksprache mit den Arbeitsgruppen weitere Änderungen vorgenommen. Die Konsensuskonferenz ist für den Sommer 2024 geplant. Im Rahmen der Konsensuskonferenz findet eine strukturierte Konsensfindung mithilfe eines nominalen Gruppenprozesses statt. Die jeweils erreichten Konsensstärken werden in der Leitlinie ausgewiesen (vgl. Tab. 2).

Die Geltungsdauer der Leitlinie ist auf 5 Jahre begrenzt [2]. Eine anlassbezogene frühere Aktualisierung ist möglich. Der Aktualisierungsbedarf wird regelhaft durch die Mitglieder des Arbeitskreises „Schmerz und Alter“ der Deutschen Schmerzgesellschaft e. V. geprüft.

## Externe Begleitung

Die Moderation des formalen Konsensusprozesses wird durch Expert:innen der AWMF begleitet werden. Vor der Veröffentlichung im AWMF-Leitlinienregister erfolgt eine formale Prüfung der Dokumente durch die AWMF.

## Ethische Aspekte der Leitlinie

Ältere und insbesondere Menschen mit kognitiven Einschränkungen (z. B. Demenz) laufen Gefahr, dass ihre Schmerzen unzureichend erkannt und behandelt werden [1]. Dabei besteht ein Zusammenhang zwischen der Schmerzintensität und einer verringerten Lebensqualität [17]. Da geriatrische Patient:innen eine hohe Vulnerabilität aufweisen, ist ihnen ein besonderer Schutz zu gewährleisten [34]. Die Leitlinie hat zum Ziel, das Schmerzmanagement geriatrischer Patient:innen zu verbessern, und entspricht damit den Forderungen der Deklaration von Helsinki [34].

## Implementierung und Verbreitung der Leitlinie

Im Rahmen der Leitlinienentwicklung werden Qualitätsindikatoren anhand eines mehrstufigen Prozesses entwickelt. Zu Beginn werden die in den Quellleitlinien angegebenen Qualitätsindikatoren (QI) gelistet und einer ersten Sichtung unterzogen. Ebenso wird eine systematische Literaturrecherche nach (inter-)nationalen Qualitätsindikatoren durchgeführt. Nach der finalen Konsentierung der Leitlinienempfehlung werden des Weiteren die starken Empfehlungen gelistet und um die weiteren verfügbaren QI ergänzt. Darauf aufbauend wird ein Set an QI auf Grundlage der Relevanz, Wissenschaftlichkeit und Praktikabilität entwickelt [12]. Die Pilotierung der Qualitätsindikatoren ist zu einem späteren Zeitpunkt beabsichtigt.

Für die Endfassung der Leitlinie sollen eine Kurz- und Langversion entstehen. Ebenso soll eine Patient:innenversion gemeinsam mit den Patient:innenvertretungen erarbeitet werden und die wichtigsten Empfehlungen aus Sicht der Patient:innen beinhalten.

## Fazit

Die S3-Leitlinie „Schmerzmanagement bei geriatrischen Patient:innen in allen Versorgungssettings“ bildet die Grundlage für eine hohe Versorgungsqualität bei dieser besonders vulnerablen Zielpopulation. Insbesondere Menschen mit Demenz sind einem hohen Risiko für eine Fehlbehandlung ausgesetzt. Eine evidenzbasierte Leitlinie, die sich den Besonderheiten des Schmerzmanagements bei geriatrischen Patient:innen vom Screening bis hin zur Therapie widmet und dabei die setting- wie professionenübergreifenden Herausforderungen berücksichtigt, bildet dafür ein wichtiges Fundament. Die Vertretung verschiedenster Disziplinen und Professionen in der Leitliniengruppe spiegelt die Versorgungsrealität von geriatrischen Patient:innen gut wider. Dadurch soll die Umsetzung eines evidenzbasierten Schmerzmanagements bei geriatrischen Patient:innen gefördert werden.

## Data Availability

Für den vorliegenden Artikel haben wir keine Datensätze analysiert oder erstellt, da es sich hierbei um einen theoretischen Ansatz handelt. Alle weiteren Informationen finden Sie unter der AWMF-Registernummer 145-005.
